# Microwave-induced plasma reduction of Sc_2_O_3_ for sustainable Al_3_Sc alloy production: *In Situ* analysis of Al_3_Sc formation mechanisms

**DOI:** 10.3389/fchem.2025.1525997

**Published:** 2025-03-10

**Authors:** Jun Fukushima, Yuya Okawa, Tomoaki Miyazawa, Hirotsugu Takizawa, Satoshi Fujii

**Affiliations:** ^1^ Department of Applied Chemistry, Graduate School of Engineering, Tohoku University, Sendai, Miyagi, Japan; ^2^ Furuya Metal Co., Ltd, Ibaraki, Japan; ^3^ Department of Information and Communication System Engineering, National Institute of Technology, Okinawa College, Okinawa, Japan

**Keywords:** microwave induced plasma, reduction, Al_3_Sc, gas analysis, sustainable alloy

## Abstract

This study aimed to elucidate the reaction mechanism of the microwave irradiation method, a novel approach for synthesizing scandium (Sc) and aluminum (Al). Sc_2_O_3_ and Al mixed powders were exposed to Mg vapor or Mg plasma. *In situ* gas analysis and XRD analysis revealed that Al_3_Sc was formed as a result of Al pulling out Sc while Mg acted as an oxygen getter. In particular, Mg plasma was shown to directly reduce Sc_2_O_3_ and significantly increase Al_3_Sc formation due to its high-energy state. These findings highlight the crucial role of Al and Mg vapors in the synthesis of Sc-containing alloys and demonstrate that Mg plasma accelerates the reaction rate through a distinct mechanism compared to Mg vapor. This study’s outcomes are expected to contribute to the development of environmentally friendly and efficient processes for producing Sc-containing aluminum alloys using Sc_2_O_3_, a material challenging to smelt by conventional methods.

## 1 Introduction

Scandium (Sc) is a metal characterized by low specific gravity and a relatively high melting point, making it a valuable addition to metals, alloys, and ceramics for enhancing material functionality. Al-Sc alloys, including intermetallic compounds of scandium (Sc) and aluminum (Al) (Shubin et al., 2008), are expected to be low-density materials with high strength at high temperatures. Al alloys with small amounts of Sc are uniquely advantageous as they combine light weight with exceptional strength (Shubin et al., 2008). For example, Sc-doped Al alloys exhibit significant hardening after quenching (Røyset and Ryum, 2005). This hardening was attributed to the presence of very fine equilibrium A1_3_Sc precipitates in the ordered L_12_ structure (Jo and Fujikawa, 1993), which is a type of regular atomic arrangement found in intermetallic compounds. As this case, intermetallic compounds with specific composition ratios can sometimes exhibit properties that differ from those of alloys. For example, Al-Zr-Sc and Al-Mg-Sc, alloys demonstrate anti-recrystallization effect (Røyset and Ryum, 2005) and superplastic behavior (Nieh et al., 1998), leading to excellent mechanical properties and high ductility. Additionally, nitrides of Al-Sc alloys exhibit high thermal conductivity and are promising as electronic component materials due to their wide-gap semiconductor characteristics (Talley et al., 2018; Liauh et al., 2016; Berkok et al., 2013). These Sc-doped intermetallic compounds and ceramics are anticipated to serve as functional materials in diverse applications, including aerospace, automotive, dielectric, semiconductor, and MEMS devices.

Although scandium (Sc) is classified as a rare earth element, it is relatively abundant in the Earth’s crust and is found in various ores. However, its dispersion in the Earth’s crust and the challenges associated with smelting Sc_2_O_3_, one of the most thermodynamically stable oxides, make its reduction a complex and energy-intensive process. A common method of Sc smelting involves converting Sc_2_O_3_ to ScF_3_ and reducing it with Ca (Daane and Hampel, 1971). This process is inherently complicated, costly, and environmentally unfriendly due to the involvement of fluorides. In recent years, alternative methods for directly preparing alloys from Sc sources, bypassing the need to produce Sc metal before forming Al-Sc alloys, have been actively explored. For example, the electrochemical production of Al-Sc alloys has been investigated by several groups (Harata et al., 2009; Liu et al., 2019; Liao et al., 2024). Additionally, thermal reduction methods utilizing collector metals have been reported. For example, Fisher et al. described a method for extracting Sc-Zn alloys using ScF_3_ as a starting material, with Zn serving as the collector metal (Spedding et al., 1960; Fischer et al., 1937). Similarly, Harata et al. obtained Sc-Al alloys from Sc_2_O_3_ at 1,000°C by employing Al as a collector metal during Ca thermal reduction (Harata et al., 2008). Furthermore, Fujii et al. successfully obtained Al-Sc alloys via microwave irradiation without the use of molten salt (Fujii and Fukushima, 2023; Fukushima et al., 2024; Fujii et al., 2020). The direct production of Al-Sc alloys from Sc_2_O_3_ without involving fluorides, offers significant advantages in terms of cost-effectiveness and environmental impact, making it a focus of intensive research.

The synthesis of Al_3_ Sc alloys using microwave irradiation is anticipated to save energy by reducing processing time and lowering required temperatures. However, the underlying smelting mechanism remains unclear. Based on the XRD results from previous basic experiments, the crystalline phases in the samples obtained using the Mg plasma generator with a single-mode cavity were identified as Al_3_Sc, MgO, and an unreacted raw material phase; notably, Al_2_O_3_ was not detected (Fujii and Fukushima, 2023). Consequently, the overall reaction equation, excluding unreacted raw materials, is proposed as follows:

$${\text{Sc}}_{2}O_{3}\;\left(s\right)+6\text{Al }\left(s\text{ or }l\right)+3\text{Mg }\left(g\text{ or plasma}\right)=2\;{\text{Al}}_{3}\text{Sc}+3\text{MgO}$$ (1)

The Mg consumption during the reaction, determined from the pre-and post-experimental weights, was nearly identical to the Mg requirement calculated using Equation 1 for the synthesis of Al_3_Sc. This finding confirms the plausibility of Equation 1. According to the Sc-Al phase diagram, equilibrium intermetallic compounds such as Al_3_Sc, Al_2_Sc, AlSc, and AlSc_2_ were formed, with the thermodynamic equilibrium phase being Al_3_Sc (Røyset and Ryum, 2005). However, while Al_3_Sc was identified as the primary product, the driving force for the reduction of Sc_2_O_3_ remained unclear. Although the experiment was conducted under vacuum conditions, the base pressure was approximately 2 Pa. Based on the Ellingham diagram, Al and Mg cannot act as Sc_2_O_3_ reductants at the experimental temperature range (Chase, 1998). The sample temperature during the microwave process did not exceed 1,000°C (Fujii et al., 2020), although this measurement was uncertain as it was based on crucible temperatures from a previous study. These observations suggest that the reduction mechanism of Sc_2_O_3_ does not involve a simple reduction to Sc. The liquidus line of the Al_3_Sc intermetallic compound is 1,050°C, yet the experimental temperature remained below 700°C. This indicates that the formation of Al_3_Sc intermetallic compounds occurred through a mechanism distinct from the precipitation of Al_3_Sc from molten Al-Sc alloy during cooling. On the other hand, Mg in its plasma state likely contributed to the reduction of Sc_2_O_3,_ possibly due to the high-energy characteristics of the plasma, though the specific mechanism remains unclear. Moreover, the simultaneous heating of Sc_2_O_3_–Al pellets and the introduction of Mg plasma were performed in a single microwave furnace; consequently, the complexity of the system precluded a clear elucidation of the driving forces of the chemical reaction (Fujii et al., 2020).

To elucidate the reaction mechanism of Al_3_Sc alloy formation, it is essential to determine the role of Al, the contribution of Mg, and the effect of Mg plasma under conditions that exclude the influence of microwave electric and magnetic fields on oxide reduction (Fukushima et al., 2012; Mizuno et al., 2021). In this study, the mechanism of Al_3_Sc formation was investigated by heating only mixed Sc_2_O_3_ + Al powder pellets, and the effect of Mg vapor on these pellets was evaluated using *in situ* gas analysis. Additionally, the influence of Mg plasma was assessed using *in situ* gas analysis within an experimental system where Mg plasma was applied to Sc_2_O_3_ -Al pellets heated in an electric furnace. This setup employed a technique that maintained Mg plasma for an extended duration and generated the plasma outside the microwave cavity.

## 2 Experimental procedure

### 2.1 Sc_2_O_3_ + tube furnace heating of Al pellets

**FIGURE 1:**
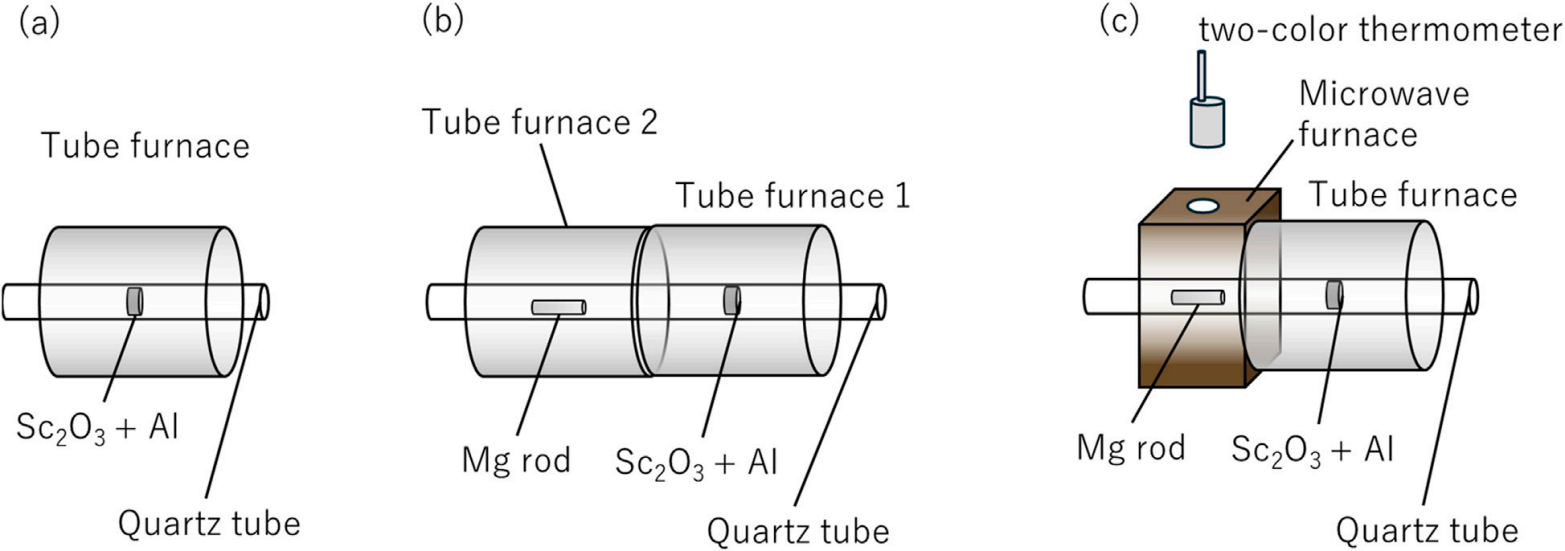
**(A)** Sc_2_O_3_ + tube furnace heating of Al pellets. **(B)** Mg vapor effect on Sc_2_O_3_ +Al pellets heated by tube furnace heating. **(C)** Mg plasma effect on Sc_2_O_3_ +Al pellets heated using tube furnace heating.

Sc_2_O_3_:Al = 1:10 mol of raw powder (Sc_2_O_3_: 4 N up, Hunan Oriental Scandium Co., Ltd. Al: 4 N up, 150 µm under, Kojundo Chemical Lab. Co., Ltd.) was mixed to produce 1.5 g of 13 mm⌀ O.D. pellet. The pellets were set in a ⌀18 mm quartz tube, which was inserted into a tube furnace (Figure 1A). A vacuum atmosphere (<2 Pa) was achieved using an oil rotary pump. The pellets were heated to 700°C with a heating duration of 30 min, followed by a holding period of 30 min. *In situ* gas analysis was conducted during the heating process using a differential pumping system (Qulee with YTP-H, ULVAC, Inc.), which comprised a quadrupole mass spectrometer and a turbomolecular pump. After the heating process, the heater power was turned off, and the samples were allowed to cool naturally. The cooled samples were subsequently ground, and XRD measurements were performed to analyze the crystalline phases.

### 2.2 Mg vapor effect on Sc_2_O_3_ +Al pellets heated by tube furnace heating

The same pellets as in (a) were used. As the Mg source, about 1.2 g of ⌀10 mm Mg rods were cut out. As shown in Figure 1B, two tube furnaces were used, with Sc_2_O_3_ +Al pellets placed on the vacuum pump side. The pellets were subjected to thermal treatment at 700°C under identical conditions to (a), with a heating duration of 30 min and a holding period of 30 min. Mg vapor was generated in the second furnace under similar conditions, with a heating time of 30 min and a holding time of 30 min at 650°C. To observe the degassing behavior from the pellets and evaluate the effect of Mg vapor on the system, the heating of Mg was initiated 5 min after the start of Sc_2_O_3_ + Al pellet heating.

A vacuum atmosphere (<2 Pa) was maintained using an oil rotary pump throughout the experiment. *In situ* gas analysis was conducted using a differential pumping system (Qulee with YTP-H, ULVAC, Inc.), comprising a quadrupole mass spectrometer and a turbomolecular pump.

After heating, the heater was turned off, and the samples were allowed to cool naturally. The cooled samples were crushed, and XRD measurements were conducted to analyze the crystalline phases.

### 2.3 Mg plasma effect on Sc_2_O_3_ +Al pellets heated using tube furnace heating

The same pellets as in (a) were used. As the Mg source, about 1.2 g of ⌀10 mm Mg rods were cut out. The tube furnace and microwave cavity were arranged as shown in Figure 1C with Sc_2_O_3_ +Al pellets on the vacuum pump exhaust side. A vacuum atmosphere (<2 Pa) was maintained using an oil rotary pump. The pellets were heated to 700°C as in conditions (a) and (b), with a heating duration of 30 min and a holding time of 30 min. TM110 single-mode microwave cavity (irradiated at the maximum position of the H-field) was used to increase the Mg temperature to approximately 650°C for 30 min and to generate Mg plasma. Microwave output was manually controlled to keep the temperature constant at less than 150 W. The temperature of the Mg rod was measured using a two-color thermometer through the hole at the top of the cavity.

*In situ* gas analysis was conducted using a differential pumping system that combined a quadrupole mass spectrometer (QMG250 PrismaPro, Hakuto Co., Ltd.) and turbo molecular pump (HiCube30 Eco, Hakuto Co., Ltd.). The obtained samples were ground, and XRD measurements were conducted to analyze the crystalline phases.

## 3 Results

### 3.1 *In-situ* gas analysis results during heating

**FIGURE 2:**
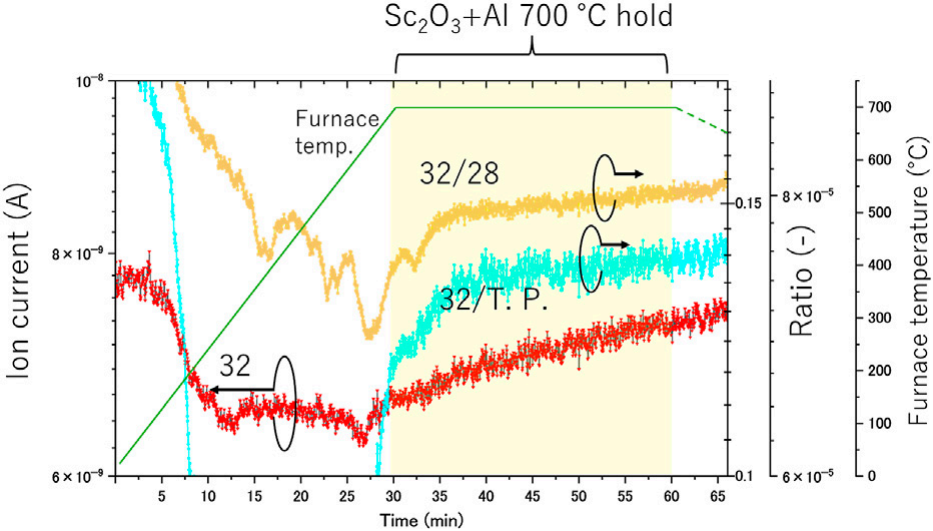
*In-situ* gas analysis during tube furnace heating of the Sc_2_O_3_ + Al pellet.

The *in situ* gas analysis during tube furnace heating of the Sc_2_O_3_ +Al pellet is presented in Figure 2. The “32” in the figure shows the ion current corresponding tor a mass number of 32. “32/28” indicates the ratio of the ion current at mass number 32 to that at mass number 28, and “32/T. P.” represents the ratio of the ion current at mass number 32 to the total pressure. It was difficult to conduct an experiment in which quantitative values could be discussed owing to the limitations of the experimental system. Therefore, we will not discuss quantitative values for gas analysis but only discuss the reaction mechanism based on the trend of changes in the gas partial pressure.

The horizontal axis shows time, with the tube furnace reaching a temperature of 700°C after approximately 30 min of measurement. The trend of mass number 32 shows that the ion current decreases as the tube furnace temperature rises. Although there is a slight increase after approximately 10 min, the value remains relatively unchanged thereafter. The “32/28” ratio remained constant for atmospheric compositions, but as the furnace temperature increased, the “32/28” ratio decreased, indicating that the nitrogen adsorbed in the quartz tube was desorbed. As mass number 32 increased in the latter half of the heating process, the “32/28” ratio also began to rise, and this trend persisted during the temperature-holding phase. If the increase in mass number 32 had been caused solely by the desorption of adsorbed air in the system, “32/28” and “32/T.P.” would not have increased. However, their observed increase suggests that the rise in mass number 32 resulted from a cause other than the desorption of adsorbed air. Therefore, the increase in mass number 32, corresponding to oxygen, indicates that oxygen was released from the Sc_2_O_3_ +Al pellet during heating and holding at 700°C.

**FIGURE 3:**
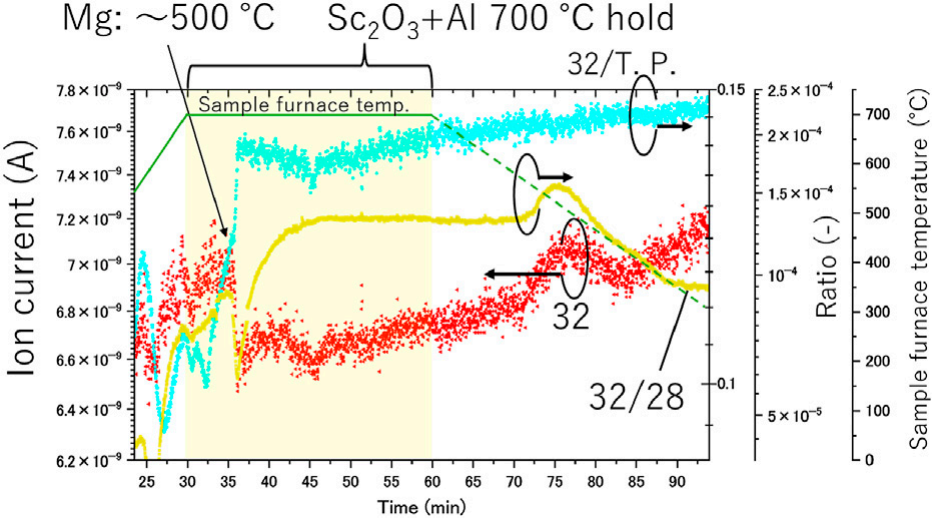
*In-situ* gas analysis during Experiment (b).

The results of the *in situ* gas analysis during Experiment (b) are shown in Figure 3. As indicated in the experimental method, heating of the Mg rod in the tube furnace began after the Sc_2_O_3_ +Al pellet heating, resulting in the pellets reaching 700°C first. After 20 min of heating the Sc_2_O_3_ +Al pellet, it was verified that the mass numbers 32, 32/28, and 32/T.P. increased, similar to the observations in Experiment (a). These values continued to rise until the temperature of the tube furnace for Mg heating reached approximately 500°C. When the tube furnace for Mg heating reached approximately 500°C, the mass number of 32 and 32/28 decreased rapidly, while 32/T.P. increased. It is known that under vacuum, the vapor pressure of Mg increases significantly from approximately 450°C. Since the Mg rod was vacuum-insulated in the quartz tube, its temperature rose slightly slower than that of the surrounding surface. Once the Mg temperature exceeded 450°C, Mg began to evaporate and deposit on the quartz tube wall. During this evaporation process, Mg solidified while adsorbing oxygen and nitrogen, resulting in a verified gettering effect, as evidenced by the reduction in total system pressure and the decrease in 32/T.P. This effect was maintained for approximately 10 min. However, the partial pressure of oxygen increased after 40 min, corresponding to the complete evaporation of the Mg rods. This was confirmed by the observation that all Mg rods had fully evaporated by the end of the experiment. Based on the results of Experiment(a) and the observations of the tube furnace for Mg heating below 500°C, it can be inferred that oxygen continued to be released from the Sc_2_O_3_ +Al pellet while holding at 700°C. Despite this release, the system’s oxygen partial pressure decreased significantly due to the gettering effect of Mg. Thus, in a system where Mg vapor is present, the oxygen partial pressure of the system decreases significantly despite the release of oxygen from the Sc_2_O_3_ +Al pellet, highlighting the substantial gettering effect of Mg on the system’s oxygen partial pressure.

**FIGURE 4:**
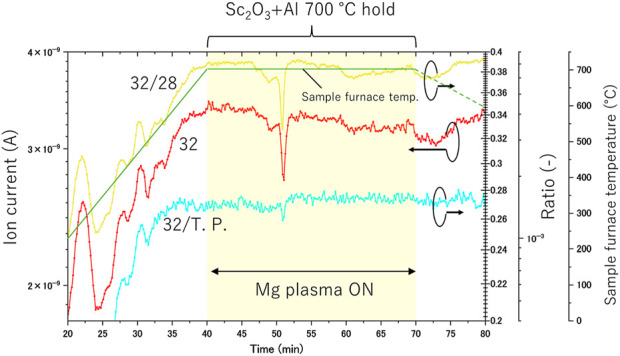
*In-situ* gas analysis during Experiment (c).

The *in situ* gas analysis results for the Mg plasma are presented in Figure 4. Similar to the results of Experiments (a) and (b), the values of 32, 32/28, and 32/T.P. increased before the Sc_2_O_3_ +Al pellet reached 700°C, confirming the release of oxygen from the pellet. A decrease in the oxygen partial pressure in the system was observed when the Mg plasma was applied 40 min after the start of mass spectrometry. However, a rapid decrease in the oxygen partial pressure was not detected, and the rate of oxygen partial pressure reduction per unit time was smaller than that in Experiment (b). When Mg plasma is generated using microwaves, it is estimated that the amount of Mg evaporation is lower than during Mg rod evaporation in tube furnace heating. This is because a significant portion of microwave energy is consumed for maintaining the Mg plasma rather than heating the Mg itself. Therefore, the Mg rods were maintained for approximately the same time as in Experiment (b), they were found to remain unconsumed after the experiment. This observation aligns with the reduced decrease in oxygen partial pressure compared to the Mg vapor generation experiment. In short, the effect of the Mg plasma by microwave irradiation on the oxygen partial pressure decrease was small compared to the effect of Mg evaporation by the tube furnace.

### 3.2 XRD results of post-experiment samples

**FIGURE 5:**
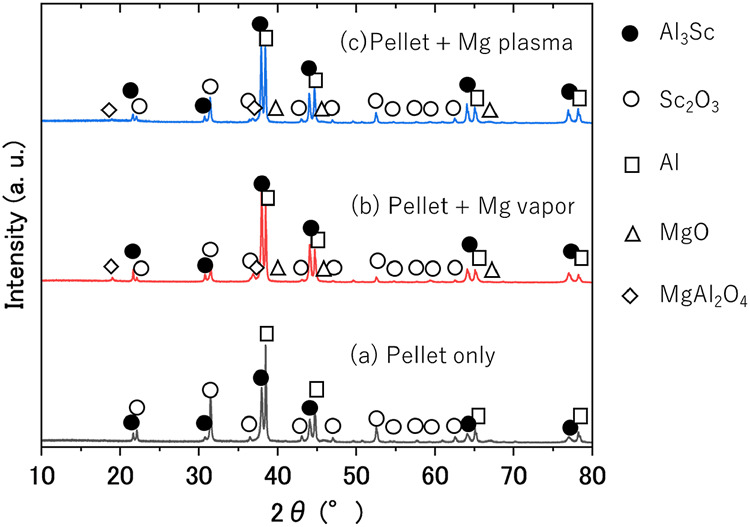
XRD patterns of the samples obtained after Experiment (a), (b), and (c).

**FIGURE 6:**
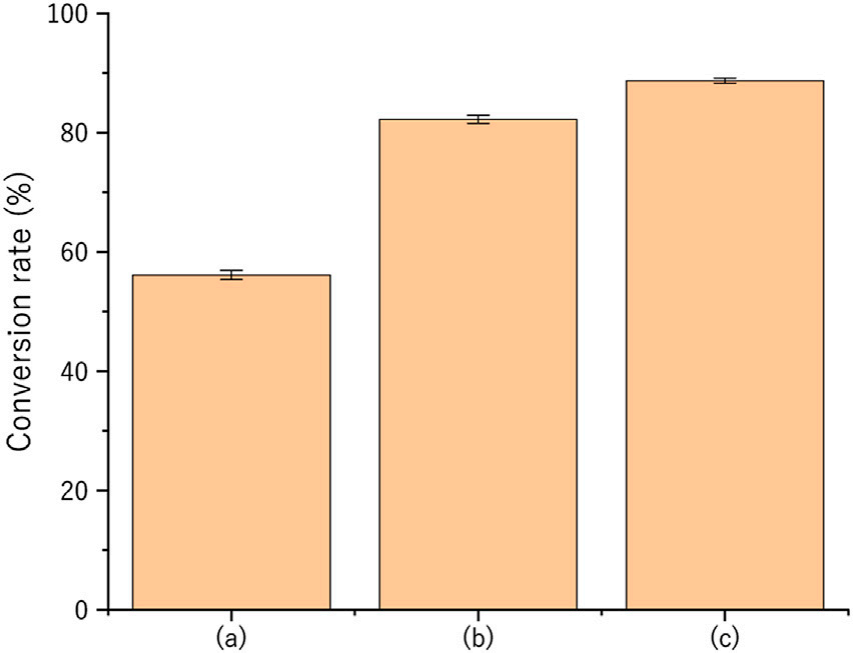
Conversion rates of the sample (a), (b), and (c).

Figure 5 shows the XRD patterns of the post-experiment samples. In pattern (a), the formation of Al_3_Sc was observed, along with the presence of unmatched raw materials, Al and Sc_2_O_3_. This indicated that Sc-Al alloys can be synthesized by heating under vacuum at 700°C for 30 min, even in a system where Mg does not participate. Notably, no peaks corresponding to Al_2_O_3_ were detected. The XRD pattern in (b) reveals the formation of MgO and MgAl_2_O_4_ in addition to Al_3_Sc. MgO is known to be reduced by Al at high temperatures to form MgAl_2_O_4_ spinel (4MgO + 2Al → 3Mg + MgAl_2_O_4_) (Yang et al., 2006). As in pattern (a), no peaks corresponding to Al_2_O_3_ were observed. The XRD pattern of (c) is similar to that of (b), with peaks corresponding to MgO and MgAl_2_O_4_ observed alongside those of Al, Sc_2_O_3_, and Al_3_Sc. Similar to the previous patterns, no formation of Al_2_O_3_ phases was detected. From this XRD pattern, the respective mass fractions were calculated using the RIR method to obtain the conversion ratio, which is the conversion rate from Sc_2_O_3_ to Al_3_Sc. The results are shown in Figure 6. The conversion rates for (a), (b), and (c) are 57%, 82%, and 89%, respectively. Even when considering the uncertainty of the wt% values obtained using the RIR method, the values showed a significant difference. The highest yield of Al_3_Sc was obtained when Mg plasma was applied. These *in situ* experiments were performed only once. On the other hand, conversion rates of more than 90% were repeatedly obtained under magnesium plasma using a multi-mode microwave process for 60 min, as shown in Supplementary Figure S1. In the following section, the reasons why such high conversion rates can be achieved under Mg plasma are considered.

## 4 Discussion

A series of results related to Experiment (a) demonstrates that Al_3_Sc can be obtained simply by heating a mixed powder of Sc_2_O_3_ and Al under a vacuum. During the reaction, oxygen was released, and no Al_2_O_3_ was detected in the sample after the experiment. These observations form the basis for discussing the mechanism by which Al_3_Sc was formed in Experiment (a). First, based on the Ellingham diagram and the absence of Al_2_O_3_ formation, it is unlikely that aluminum in its solid or liquid phases directly reduced Sc_2_O_3_. The fact that oxygen was released from the pellet indicates that Sc_2_O_3_, the only oxide in the system, underwent reduction. However, according to the Ellingham diagram, Sc_2_O_3_ being highly stable is unlikely to be reduced to Sc metal under the experimental conditions. From the above, the following elementary reactions are proposed:

$$Sc_{2}O_{3}{\to \text{Sc}}_{2}O_{3-\delta }+\delta {/2O}_{2\;}\left(g\right)$$ (2)

$${\text{Sc}}_{2}O_{3-\delta \;}+3\delta \text{Al}\to \delta {\text{Al}}_{3}\text{Sc}+\left(1‐\delta \right){\text{Sc}}_{2}O_{3\;}$$ (3)

Oxygen is released according to Equation 2, and the formation of Al_3_Sc proceeds via Equation 3. In this mechanism, aluminum acts as a collector metal, extracting Sc from the weakly reduced scandium oxide. Although the precise amount of oxygen loss(δ) has not been quantified, δ is expected to be small due to the high stability of Sc_2_O_3_. However, the conversion ratio of Al_3_Sc exceeds 50%; suggesting that the driving force of Al as a collector metal is significant in this system.

Next, the effect of Mg vapor is discussed. The XRD results indicate the formation of MgO, but it is unlikely that Mg, which is considered to have a lower reducing power than Al, directly reduced Sc_2_O_3_. *In-situ* gas analysis revealed that the partial pressure of oxygen in the system decreased immediately after the generation of Mg vapor, This observation suggests that the oxygen released via Equation 2 reacts with Mg vapor to form oxides, as shown in Equation 4

$$\delta /2O_{2\;}\left(g\right)+\delta \text{Mg }\left(g\right)\to \delta \text{Mg}O\;\left(s\right)$$ (4)

The reaction in Equation 4 causes the equilibrium in the overall reaction in Equation 1 to shift to the right, increasing the amount of Al_3_Sc produced. Due to this oxygen-gettering effect, the conversion ratio, which was 57% in the absence of Mg vapor, increased to 82%.

Finally, the effect of the Mg plasma is discussed. The XRD results revealed that the maximum conversion ratio reached 89%, indicating that the reaction is accelerated in the presence of Mg plasma compared to the Mg vapor. This demonstrates that Mg plasma enhances the reaction rate more effectively than Mg vapor. While Equation 4 suggests a possible effect of Mg plasma on lowering the oxygen partial pressure in the system, the *in situ* gas analysis results indicate that although the oxygen partial pressure was reduced, the oxygen partial pressure reduction per unit time was smaller than that in the Mg vapor experiment. These observations suggest that the effects of Mg vapor and Mg plasma on the reaction system differ significantly. From the formation of MgO on the right side of the overall reaction, it is strongly inferred that a portion of the Mg plasma contributes to the direct reduction of scandium oxide, thereby promoting the reaction. This can be represented by Equation 5:

$${\text{Sc}}_{2}O_{3}\left(s\right)+\text{Mg}\left(\text{plasma}\right)\to {\text{Sc}}_{2}O_{3-\delta }+\delta \text{MgO}$$ (5)

This finding is consistent with the results of a previous study (Fujii et al., 2020). From the above, Al_3_Sc smelting is achieved through a mechanism where aluminum acts as a collector metal, extracting Sc from slightly reduced Sc_2_O_3_, Mg vapor further promotes the reaction by decreasing the system’s oxygen partial pressure, while the plasma conversion of Mg vapor partially contributes to the direct reduction of scandium oxide, thereby enhancing the reaction.

## 5 Conclusion

This study investigated the reaction mechanism involved in the synthesis of Al_3_Sc alloys using Mg plasma applied to Sc_2_O_3_-Al mixtures via microwave irradiation, analyzed through *in situ* gas analysis. The findings revealed that Al acts as a collector metal, extracting Sc from slightly reduced Sc_2_O_3_, while Mg vapor promotes the reaction by functioning as an oxygen getter. Furthermore, the direct reduction of Sc_2_O_3_ by Mg in the plasma state was confirmed, leading to an increased yield of Al_3_Sc compared to the Mg vapor case. These results emphasize the critical role of Al and Mg in the development of novel synthesis methods for Sc-containing alloys and demonstrate that Mg plasma enables smelting via a reaction pathway distinct from conventional methods. The outcomes of this study will contribute to the development of manufacturing processes for Sc-containing alloys, addressing the limitations of traditional methods that impose a large environmental burden. Moreover, these findings open the possibility of developing new alloy formation routes by leveraging the appropriate interactions of collector metals, metal vapors, and metal plasma, even with other difficult-to-reduce oxides as starting materials.

## Data Availability

The original contributions presented in the study are included in the article/supplementary material, further inquiries can be directed to the corresponding author.

## References

[B1] Berkok H. Tebboune A. Saim A. Belbachir A. H. (2013). Structural and electronic properties of ScxAl1−xN: first principles study. Phys. B Condens Matter 411, 1–6. 10.1016/j.physb.2012.11.035

[B2] Chase M. W. (1998). NIST-JANAF thermochemical tables. Forth Edn. (American Institute of Physics).

[B3] Daane A. H. (1971). Rare metals handbook. Editor Hampel C. A. (USA: Krieger Publishing Company), 441.

[B4] Fischer W. Brunger K. Grieneisen H. (1937). Über das metallische Scandium. Z Anorg. Allg. Chem. 231, 54–62. 10.1002/zaac.19372310107

[B5] Fujii S. Fukushima J. (2023). Metal ion plasma generation under strong magnetic field in microwave resonator. AIP Adv. 13 (1), 015320. 10.1063/5.0134071

[B6] Fujii S. Suzuki E. Inazu N. Tsubaki S. Fukushima J. Takizawa H. (2020). Microwave irradiation process for Al–Sc alloy production. Sci. Rep. 10 (1), 2689. 10.1038/s41598-020-59664-2 32060366 PMC7021902

[B7] Fukushima J. Fujii S. Hirotsugu T. (2024). Rapid synthesis of ceramics by microwave solid-state process and application of plasma carbon recycling. J. Jpn. Soc. Colour. Mater 97 (6), 176–180. 10.4011/shikizai.97.176

[B8] Fukushima J. Kashimura K. Takayama S. Sato M. (2012). Microwave-energy distribution for reduction and decrystallization of titanium oxides. Chem. Lett. 41 (1), 39–41. 10.1246/cl.2012.39

[B9] Harata M. Nakamura T. Yakushiji H. Okabe T. H. (2008). Transactions of the institutions of mining and metallurgy, section C: mineral processing and extractive metallurgy. 117 (2), 95.

[B10] Harata M. Yasuda K. Yakushiji H. Okabe T. H. (2009). Electrochemical production of Al–Sc alloy in CaCl2–Sc2O3 molten salt. J. Alloys Compd. 474 (1–2), 124–130. 10.1016/j.jallcom.2008.06.110

[B11] Jo H.-H. Fujikawa S. (1993). Kinetics of precipitation in Al-Sc alloys and low temperature solid solubility of scandium in aluminium studied by electrical resistivity measurements. Mater. Sci. Eng. 171, 151–161. 10.1016/0921-5093(93)90401-y

[B12] Liao C. Que L. Fu Z. Deng P. Li A. Wang X. (2024). Research status of electrolytic preparation of rare earth metals and alloys in fluoride molten salt system: a mini review of China. Met. (Basel) 14 (4), 407. 10.3390/met14040407

[B13] Liauh W. J. Wu S. Huang J. L. Lii D. F. Lin Z. X. Yeh W. K. (2016). Microstructure and piezoelectric properties of reactively sputtered highly C-axis ScxAl1-xN thin films on diamond-like carbon/Si substrate. Surf. Coat. Technol. 308, 101–107. 10.1016/j.surfcoat.2016.06.097

[B14] Liu X. Xue J. Guo Z. Zhang C. (2019). Segregation behaviors of Sc and unique primary Al3Sc in Al-Sc alloys prepared by molten salt electrolysis. J. Mater Sci. Technol. 35 (7), 1422–1431. 10.1016/j.jmst.2019.02.002

[B15] Mizuno N. Kosai S. Yamasue E. (2021). Microwave-based extractive metallurgy to obtain pure metals: a review. Clean. Eng. Technol. 5, 100306. 10.1016/j.clet.2021.100306

[B16] Nieh T. G. Hsiung L. M. Wadsworth J. Kaibyshev R. (1998). High strain rate superplasticity in a continuously recrystallized Al–6%Mg–0.3%Sc alloy. Acta mater 46 (8), 2789–2800. 10.1016/s1359-6454(97)00452-7

[B17] Røyset J. Ryum N. (2005). Scandium in aluminium alloys. Int. Mater. Rev. 50, 19–44. 10.1179/174328005x14311

[B18] Shubin A. B. Shunyaev K. Yamshchikov L. F. (2008). Thermodynamic Properties of Intermetallic Compounds in Al-Sc, Cu-Sc and Pb-Sc Systems. Archives Metallurgy Mater. 53 (4), 1119.

[B19] Spedding F. H. Daane A. H. Wakefield G. Dennison D. H. (1960). Transaction of the metallurgical society of AIME. vol. 218. New York, NY: American Institute of Mining, Metallurgical, and Petroleum Engineers. 608.

[B20] Talley K. R. Millican S. L. Mangum J. Siol S. Musgrave C. B. Gorman B. (2018). Implications of heterostructural alloying for enhanced piezoelectric performance of (Al,Sc)N. Phys. Rev. Mater 2 (6), 063802. 10.1103/physrevmaterials.2.063802

[B21] Yang J. Kuwabara M. Sawada T. Sano M. (2006). Kinetics of isothermal reduction of MgO with Al. ISIJ Int. 46 (8), 1130–1136. 10.2355/isijinternational.46.1130

