# Supplementary material for: Microwave-induced plasma reduction of Sc2O3 for sustainable Al3Sc alloy production: In Situ analysis of Al3Sc formation mechanisms
Source: Front Chem. 2025 Mar 10;13:1525997. doi: 10.3389/fchem.2025.1525997 (PMC11931023; doi:10.3389/fchem.2025.1525997)
Supplement: Supplementary file 1 [file DataSheet1.docx]

Suppl. Info. 1


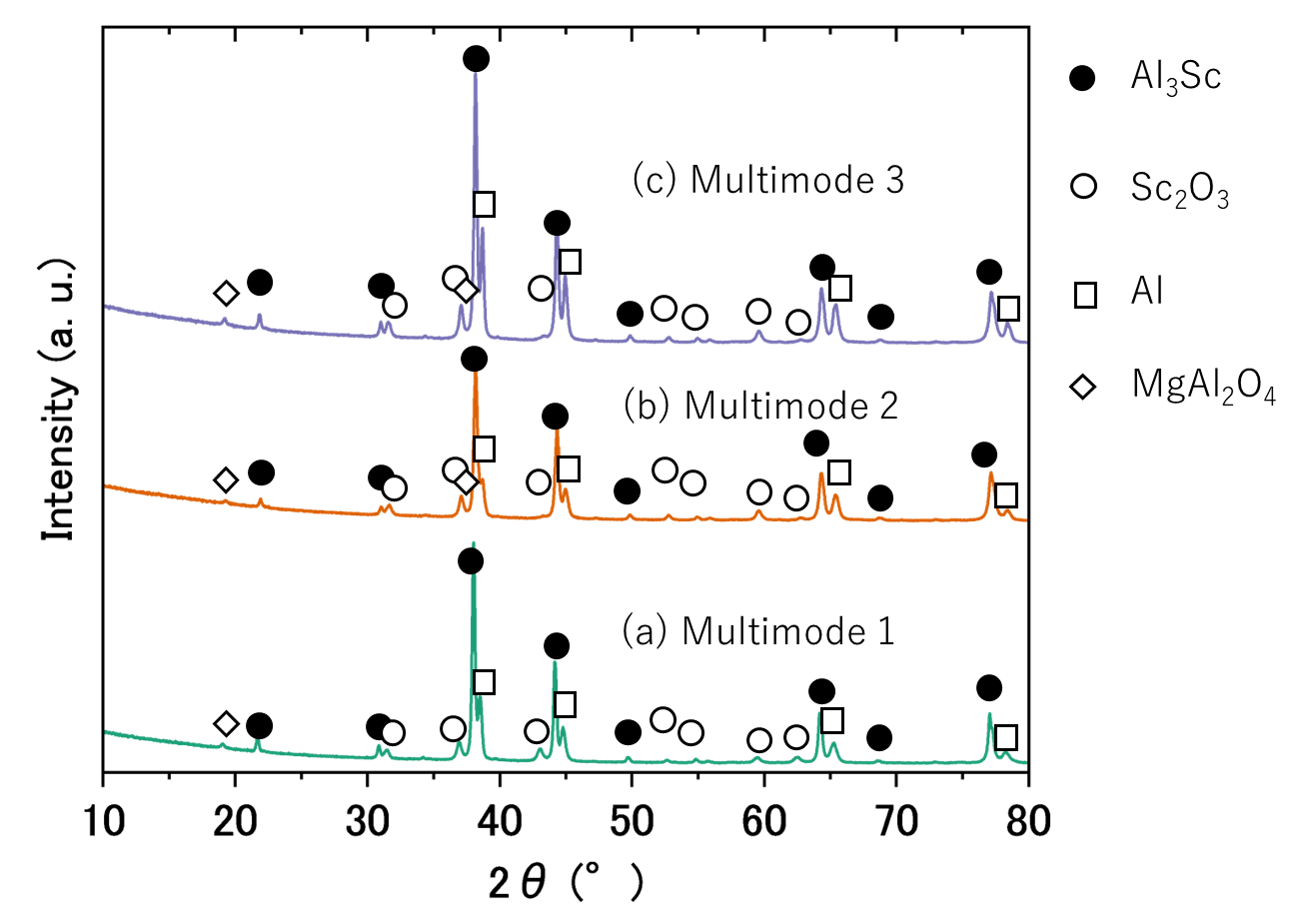


XRD patterns of the samples obtained by multimode microwave experiments. Experimental conditions were the same as in the previous study^17)^, a microwave power of approximately 300 W was applied to maintain the crucible temperature at 660 ℃ for 1 h, using a multi-mode applicator. Conversion rates were (a) 94.7%, (b) 90.3%, and (c) 90.9%, respectively.
